# Review of clinical advances in the procedural sedation application of remimazolam

**DOI:** 10.3389/fphar.2026.1740897

**Published:** 2026-02-23

**Authors:** Qiwei Wang, Shuancheng Bai

**Affiliations:** Affiliated Baotou Clinical College of Inner Mongolia Medical University, Baotou, China

**Keywords:** anesthesia, benzodiazepines, proceduralsedation, remimazolam, sedative

## Abstract

**Introduction:**

With the ongoing advancement of comfort-oriented healthcare, procedural sedation is increasingly applied in clinical practice. As a novel benzodiazepine derivative, remimazolam offers distinct advantages in procedural sedation. This paper aims to thoroughly examine its pharmacological properties and key clinical applications, providing systematic guidance for rational clinical use while promoting further adoption and development of this medication in relevant fields.

**Methods:**

Conduct a systematic analysis and summary by examining the pharmacological characteristics of remimazolam, including its mechanism of action and pharmacokinetic properties, and integrating its application scenarios, dosing regimens, and comparative efficacy data across various clinical sedation procedures.

**Results:**

Remimazolam, with its unique pharmacological advantages—such as rapid onset, short duration of action, inactive metabolites, and high safety profile—demonstrated excellent sedative efficacy and tolerability in clinical procedural sedation. It effectively meets sedation requirements across diverse procedural settings while exhibiting a low incidence of adverse reactions, thereby offering significant clinical value.

**Discussion:**

As a novel benzodiazepine derivative, remimazolam overcomes the limitations of traditional sedatives, offering a new option for procedural sedation in palliative care. Its unique pharmacologic properties ensure rapid onset, controllable duration of action, and high safety, thereby enhancing clinical procedural efficiency and patient experience. This systematic review of its clinical applications provides scientific evidence for the rational and standardized use of remimazolam in clinical practice. Future research should focus on expanding its application in more complex clinical scenarios and conducting long-term safety and efficacy studies to further promote the widespread use of remimazolam in procedural sedation.

## 1 Introduction

With the advancement of comfort-oriented healthcare, procedural sedation has gained widespread clinical application due to its controllable administration and minimal impact on vital signs. As a novel ester-type benzodiazepine, remimazolam has demonstrated significant potential across multiple clinical scenarios. This review explores the pharmacologic properties of remimazolam and its clinical applications in procedural sedation.

## 2 Pharmacological properties of remimazolam

### 2.1 Mechanism of action

Remimazolam, as a short-acting benzodiazepine, primarily exerts its effects by binding to GABAA receptors containing the α1 subunit within the central nervous system. Upon binding to GABAA receptors, remimazolam enhances the permeability of nerve cells to chloride ions. Chloride ions enter the nerve cells via concentration gradients, inducing hyperpolarization and reducing excitability. This ultimately results in sedative, hypnotic, and anxiolytic effects (Ackenbom et al., 2021).

### 2.2 Pharmacokinetic characteristics

#### 2.2.1 Absorption

Remimazolam is completely absorbed by the gastrointestinal tract after oral administration. However, due to its rapid and extensive first-pass elimination, its oral bioavailability is virtually negligible. Furthermore, the clearance rate of remimazolam and its metabolites from plasma is relatively fast after oral administration, making it difficult to produce significant sedative effects. Combined with its characteristic bitter taste, remimazolam is unsuitable for direct oral use (Pesic et al., 2020b).

Intravenous infusion of remimazolam is a commonly used clinical approach. Following injection, the drug concentration in the blood reaches its peak almost immediately and rapidly distributes to the target sites. Its average half-life ranges between 0.5 and 2 min, and the cumulative dose exhibits a dose-dependent relationship with the area under the plasma concentration-time curve (Xin et al., 2023). However, it should be noted that mixing remimazolam with acetone or lactated Ringer’s solution in the infusion line may cause precipitation. When used in combination, a lower concentration of remimazolam should be employed, and the infusion rate of Ringer’s solution should be increased to prevent precipitation (Sasaki et al., 2021).

Current research indicates that remimazolam can also be administered via the nasal route. The absorption rate of this drug through the nasal mucosa is approximately 1/50th that of intravenous administration. To achieve the same blood concentration as intravenous administration, a dose 5 to 10 times higher than the intravenous dose is required (Pesic et al., 2020a).

#### 2.2.2 Distribution

Remimazolam has a relatively small volume of distribution, approximately 50–70 L. After entering the body, it is primarily distributed in bodily fluids such as blood and interstitial fluid, with relatively little accumulation in tissues like adipose tissue. Remimazolam exhibits a high serum protein binding rate of 92%, primarily binding to site II of albumin (Freyer et al., 2019; Keam, 2020). Studies indicate that ibuprofen exhibits a strong affinity for human serum albumin, effectively displacing remimazolam bound to it. This results in a significant increase in the proportion of free remimazolam (Zhu et al., 2022). Therefore, patients taking ibuprofen may appropriately reduce the sedative dose of remimazolam.

#### 2.2.3 Metabolism and elimination

Remimazolam is primarily and rapidly hydrolyzed *in vivo* by non-specific tissue esterases in plasma, particularly CES-1, and converted into a pharmacologically inactive carboxylic acid metabolite (CNS7054) (Hu et al., 2022). Research indicates that this metabolite exhibits an affinity for GABAA receptors that is only 1/400 to 1/300 that of zolpidem (Wang et al., 2022), rendering it virtually devoid of pharmacological activity. Furthermore, its elimination follows first-order kinetics, thereby minimizing the risk of drug accumulation during prolonged or high-dose infusions (Chen et al., 2020). The elimination half-life of remimazolam is relatively short, approximately 0.7–1.1 h. Based on population pharmacokinetic modeling results from Phase I single-dose data, continuous infusion at a rate of 50 mg/h for 2–8 h maintains the average time-dependent half-life of remimazolam at a stable 7–8 min. It is particularly important to note that remimazolam is a lipophilic benzodiazepine derivative. When administered to obese patients, it may accumulate in adipose tissue, potentially prolonging the drug’s metabolic half-life (Shimamoto et al., 2022). Stöhr et al. indicated in clinical studies that no dosage adjustment is required for remimazolam in patients with mild to moderate hepatic or renal impairment. However, for patients with severe hepatic impairment, a reduced dosage of remimazolam is recommended (Stöhr et al., 2021).

## 3 Clinical application research of remimazolam in procedural sedation

### 3.1 Regional blockade

Remimazolam, as a novel benzodiazepine sedative, has demonstrated favorable performance in the clinical application of regional anesthesia sedation. In the field of combined spinal-epidural anesthesia (CSEA) sedation, Song et al. determined the median effective dose (ED50) of remimazolam for sedation in elderly patients undergoing CSEA for hip replacement surgery to be 0.212 mg/kg, with a 95% effective dose (ED95) of 0.288 mg/kg (Song and Zou, 2023). Another study involving 106 patients aged 65–90 years demonstrated that compared with propofol, remimazolam not only provided effective perioperative sedation during CSEA hip fracture surgery in the elderly but also had minimal impact on postoperative cognitive function in these patients (Duan et al., 2024). Furthermore, data comparisons revealed that the remimazolam group exhibited more stable hemodynamics and overall higher safety. In the field of spinal anesthesia sedation, Kim et al. compared the efficacy of dexmedetomidine versus remimazolam during surgical procedures under spinal anesthesia. The results demonstrated that compared to the dexmedetomidine group, the remimazolam group exhibited significant advantages in achieving target sedation depth (2.1 ± 0.8 min vs. 5.3 ± 1.2 min, P < 0.01), recovery time (12.5 ± 3.2 min vs. 22.8 ± 4.5 min, P < 0.01), reduced shivering, and enhanced patient satisfaction. These findings validate remimazolam as a potentially superior choice for surgical sedation requiring rapid recovery of consciousness (Kim et al., 2024). Lee et al. compared the efficacy and safety of remimazolam versus midazolam in preventing intraoperative nausea and vomiting (IONV) during cesarean section. They found intravenous remimazolam significantly outperformed midazolam in preventing IONV (18.3% vs. 31.7%, P = 0.045), and the remimazolam group also exhibited a lower incidence of adverse reactions such as postoperative drowsiness (Lee et al., 2024). These findings suggest that remimazolam may be a superior option for preventing IONV during cesarean section, particularly for mothers requiring rapid recovery or who are sensitive to postoperative drowsiness. However, both studies were single-center and small-sample designs, presenting certain methodological limitations. Furthermore, they failed to exclude the potential influence of confounding factors such as surgical type and patients’ underlying conditions on the study outcomes. In the field of peripheral nerve block anesthesia and sedation, Xiao et al. demonstrated through a single-center randomized controlled trial that remimazolam provided sedation comparable to propofol (96.7% vs. 93.3%, P ≤ 0.05) during ultrasound-guided transverse abdominis plane/rectus sheath block in patients undergoing abdominal tumor surgery. However, remimazolam demonstrated significant advantages in hemodynamic stability, respiratory safety, rapid recovery (5–10 min vs. 10–15 min, P ≤ 0.05), and absence of injection pain, establishing it as a safer procedural sedation agent (Xiao et al., 2023). Another study on the use of remimazolam as an adjunct sedative for brachial plexus block (BPB) in emergency hand trauma also demonstrated that compared to propofol, remimazolam achieves sedation with a lesser impact on vital signs and offers greater adjustability in sedation depth. Consequently, it is more suitable as an adjunct medication for emergency BPB (Pan et al., 2022).

### 3.2 Hysteroscopy

In recent years, remimazolam has gained widespread adoption in procedural sedation for gynecological hysteroscopic surgery due to its unique advantages. In comparison with the traditional sedative propofol, multiple studies have demonstrated that remimazolam exhibits a more pronounced sedative effect. Zhang et al. found in a single-center randomized controlled trial that the remimazolam group exhibited significantly lower incidence of hypotension (5% vs. 43.3%, P < 0.001) and hypoxemia (3.3% vs. 20.0%, P = 0.006) during sedation compared to the propofol group. Additionally, the remimazolam group exhibited shorter recovery times (7.0 ± 1.5 min vs. 10.0 ± 2.0 min, P < 0.001) and higher anesthetic satisfaction scores from surgeons. These findings suggest that remimazolam provides a safer and more comfortable surgical environment while maintaining adequate anesthetic efficacy (Zhang et al., 2021). Similarly, in a trial involving 120 subjects, Fan et al. reached comparable conclusions: remimazolam demonstrated superior hemodynamic stability compared to propofol and completely eliminated injection pain, thereby providing patients with a more favorable experience prior to sedation (Fan et al., 2023). However, the study did not provide detailed reporting on key information such as age stratification among participants or differences in baseline circulatory function, factors that may potentially influence hemodynamic outcomes. Additionally, the study did not mention whether blinded assessments were used for subjective patient experiences (e.g., injection pain), raising concerns about potential assessment bias. Zhang et al. further demonstrated through a randomized, single-blind, parallel-group controlled trial that remimazolam is a safe and reliable alternative during hysteroscopy, particularly for patient populations requiring special attention to circulatory and respiratory safety (Zhang et al., 2022).

### 3.3 Dental treatment

Dental procedures often cause anxiety, stress, and fear in patients. In recent years, the use of remimazolam in dentistry has garnered significant attention and research. A prospective exploratory study demonstrated that remimazolam monotherapy achieved a 100% success rate in sedation during outpatient third molar extraction, with no significant respiratory or circulatory effects observed during sedation and no need for additional pharmacological intervention (Oue et al., 2023). Sun et al. reported in a study on the efficacy and safety of remimazolam during tooth extraction that its use during the procedure stabilized patients’ hemodynamics, provided significant sedation, and resulted in few adverse reactions, making it a safe and reliable sedation method (Sun and Li, 2024). However, the study did not provide detailed reporting on the dose gradient of remimazolam or the criteria for monitoring sedation depth, which may have influenced the assessment of hemodynamic stability and the incidence of adverse reactions. Ba et al. compared the sedative effects of remimazolam and midazolam during impacted tooth extraction and found that remimazolam demonstrated significantly shorter onset times (1.5 min vs. 3 min, P < 0.001), awakening time (3 min vs. 5.5 min, P < 0.001), and recovery time (7 min vs. 12 min, P < 0.001). Additionally, it produced a greater reduction in postoperative dental anxiety scale (MDAS) scores (10.47 vs. 13.47, P < 0.001). This indicates that compared to midazolam, remimazolam not only effectively exerts sedative effects but also significantly enhances the efficiency of outpatient procedures and demonstrates more pronounced advantages in treating patients with dental anxiety (Ba et al., 2024). On the other hand, delayed cognitive recovery following sedation is particularly prone to occur in elderly patients undergoing tooth extraction. Liu et al. demonstrated through a prospective randomized controlled trial that remimazolam sedation significantly improves early postoperative cognitive recovery in elderly patients, accelerates the hemostasis process, and shortens their hospital stay (Liu et al., 2024). Additionally, another study examining the anesthetic efficacy of sedation during outpatient tooth extraction in elderly patients found that compared to propofol, remimazolam sedation offered advantages including more stable hemodynamics (over threefold reduction in hypotension risk), lower incidence of adverse events, and minimal impact on cognitive function (Cheng et al., 2024). Simultaneously, both physicians and patients reported higher satisfaction with the anesthetic efficacy of remimazolam, indicating its potential as a superior choice for sedation during outpatient tooth extraction in elderly patients.

### 3.4 Endoscopic retrograde cholangiopancreatography

Endoscopic retrograde cholangiopancreatography (ERCP) urgently requires an ideal sedative to enhance patient comfort, and remimazolam, as a novel sedative, demonstrates significant application potential in this field. Lee et al. compared the efficacy and safety of remimazolam versus propofol in a study of ERCP patients. Results demonstrated a 100% sedation success rate for remimazolam, along with predictable duration of action and high sedation safety. This study suggests that remimazolam may serve as a safe and effective procedural sedation regimen, offering clinicians a broader range of sedation options. This confirms remimazolam as a safe and effective sedation option capable of replacing propofol, offering clinicians a broader range of sedation choices (Lee J. et al., 2023). Additionally, another randomized controlled clinical trial demonstrated that patients receiving remimazolam during selective ERCP experienced a significantly lower risk of respiratory adverse events under deep sedation compared to the propofol group (9.6% vs. 15.7%, P = 0.04), while also exhibiting superior hemodynamic stability (Dong et al., 2023). However, the study did not report key details such as the duration of surgical procedures or the proficiency level of endoscopists, factors that may influence the incidence of respiratory adverse events. Additionally, the lack of a blinded study design may introduce subjective bias in the investigators’ assessment of adverse events. In elderly patients, Xin et al. compared the effects of remimazolam versus propofol on hemodynamics and early postoperative recovery quality during ERCP in frail elderly patients. Results showed that compared with propofol, remimazolam significantly reduced intraoperative hypotension (24.5% vs. 43.6%, P = 0.036), bradycardia (5.7% vs. 18.2%, P = 0.046), and hypoxemia (13.2% vs. 30.9%, P = 0.027) (Xin W.D. et al., 2024). Particularly for elderly patients with reduced circulatory reserve, remimazolam provided more stable hemodynamics and reduced the risk of adverse events.

### 3.5 Bronchoscope

To date, numerous scholars have explored the efficacy of using remimazolam for sedation during bronchoscopy. Zhou et al. conducted a meta-analysis to evaluate the safety and efficacy of remimazolam for patient sedation during flexible bronchoscopy. The results indicated that remimazolam demonstrated comparable sedation success rates to conventional agents (e.g., propofol, dexmedetomidine, midazolam) while exhibiting weaker respiratory and circulatory depressant effects, thereby offering superior safety (Zhou et al., 2024). However, this meta-analysis primarily focused on patients with conventional risk profiles, lacking subgroup analyses for high-risk patients (ASA III/IV), those with airway narrowing, or respiratory insufficiency, thereby limiting the generalizability of its findings. Zhang et al. found in a study of bronchoscopy with spontaneous breathing that remimazolam combined with alfentanil was more effective than propofol in reducing the incidence of respiratory depression during the procedure (13.5% vs. 39.6%, P < 0.001), and significantly reduced postoperative dizziness and abdominal distension (P < 0.05) (Zhang et al., 2023). On the other hand, Chen et al. compared the efficacy and safety of remimazolam versus dexmedetomidine in outpatient bronchoscopy. The results showed no significant differences between the two groups in terms of efficacy and safety indicators. However, the remimazolam group demonstrated significantly shorter times to onset of anesthesia, full recovery, and discharge (approximately 15%–25%, P < 0.05) (Chen et al., 2022). This suggests that remimazolam may be a more ideal procedural sedative for outpatient bronchoscopy.

### 3.6 Gastrointestinal endoscopy

With the growing demand for comfortable medical care, endoscopic procedures are increasingly becoming the mainstream choice. In the field of painless gastroscopy, Zhu et al. compared the sedative effects of different doses of remimazolam versus propofol during gastroscopy. found that the high-dose remimazolam group (0.2 mg/kg) demonstrated not only a higher sedation success rate but also superior safety compared to both the propofol group (1.5 mg/kg) and the low-dose remimazolam group (0.15 mg/kg) (P < 0.001) (Zhu et al., 2024). Similarly, a meta-analysis demonstrated that remimazolam can be safely used for gastroscopy, with lower incidence rates of respiratory depression, bradycardia, injection pain, and dizziness compared to propofol (An et al., 2024). However, this meta-analysis has certain limitations. The included studies primarily involved short-term follow-up (limited to the examination period and immediate postoperative period), lacking long-term safety data (such as effects on cognitive function and risks of drug dependence). In the field of colonoscopy, Shi et al. demonstrated that during colonoscopy procedures, both the remimazolam group (0.2 mg/kg) and the propofol group (0.2 mg/kg) achieved a 100% sedation success rate. However, the remimazolam group exhibited superior hemodynamic stability and fewer adverse effects (Shi et al., 2024). Additionally, studies by Rex et al. demonstrated that remimazolam exhibited favorable safety profiles during colonoscopy in high-risk patients classified as ASA III/IV (American Society of Anesthesiologists Physical Status Classification) III/IV high-risk patients undergoing colonoscopy, with a significantly higher procedure success rate compared to placebo (87.1% vs. 0%, P < 0.001) and midazolam (87.1% vs. 13.3%, P < 0.001), fully demonstrating remimazolam’s safety and reliability for procedural sedation in high-risk patients (Tan et al., 2022).

### 3.7 Others

Remimazolam is not only widely used in the aforementioned common procedural sedation operations but also demonstrates new exploration and application potential in other fields. In facial plastic surgery, Huang et al. found that during intubation-free anesthesia for facial procedures, remimazolam demonstrated comparable sedative effects to propofol, but with a significantly lower incidence of respiratory depression (P < 0.05). This finding strongly supports remimazolam as a safer sedative agent (Huang et al., 2024). Regarding sedation in the ICU, Tang et al. investigated the efficacy of remimazolam in maintaining sedation among patients on prolonged mechanical ventilation. Compared with propofol (1.98 mg/kg/h), remimazolam (0.18 mg/kg/h) was safe and effective for mild-to-moderate sedation in these patients. Furthermore, remimazolam may offer metabolic advantages for patients with hepatic or renal impairment (Tang et al., 2022). In terms of medical treatment, Chen Lingling et al. compared the sedative effects of remimazolam and midazolam during cardiac synchronized electrical cardioversion procedures. Results showed that remimazolam demonstrated faster onset of action (44.9 s vs. 58.5 s, P < 0.05) shorter postoperative recovery time (16.2 min vs. 22.3 min, P < 0.05), and faster orientation recovery (14.0 min vs. 27.4 min, P < 0.05) (Chen et al., 2024). This indicates that remimazolam is more suitable than midazolam as a sedative agent in scenarios requiring rapid recovery and cognitive restoration. In pediatric care, a study compared the sedative effects of remimazolam versus dexmedetomidine during cardiac ultrasound examinations in infants and young children. Results showed that compared to 2 μg/kg dexmedetomidine administered via nasal drip, intravenous administration of 0.3 mg/kg remimazolam resulted in shorter onset and recovery times, as well as a higher sedation success rate (Yang et al., 2021). This finding also suggests that intravenous remimazolam may represent a more effective sedation regimen. In summary, remimazolam demonstrates potential applications across diverse clinical settings including facial plastic surgery, ICU sedation, cardioversion, and pediatric care. Its advantages in respiratory safety, metabolic profile, and rapid recovery warrant attention. However, current research remains limited by multiple constraints: most studies employ single-center, small-sample designs with insufficient representativeness, resulting in restricted generalizability.

## 4 Adverse reactions and their prevention and treatment

In existing research and trials, the superiority of remimazolam has been preliminarily demonstrated. However, during the study period, certain adverse drug reactions were observed, including symptoms such as hypotension, respiratory depression, dizziness, and nausea. Additionally, reports of other related adverse events have emerged during clinical application. For example, multiple reports indicate that mixing remimazolam with Ringer’s solution may cause precipitation, with higher remimazolam concentrations and greater pH values increasing the likelihood of precipitation formation. Therefore, when combining the two, a low concentration of remimazolam should be used while increasing the infusion rate of Ringer’s solution to prevent precipitation (Sasaki et al., 2021; Matsuo et al., 2021; Yoshida et al., 2021). Some studies have also indicated that the incidence of allergic reactions to remimazolam is approximately 0.08%–0.15%, which is lower than that of midazolam (0.12%–0.21%) but more severe (Cinotti, 2023; Lee S. et al., 2023). Allergic reactions may manifest as hypotension, tachycardia, rash, and other symptoms. In severe cases, they can progress to anaphylactic shock and even carry a risk of cardiac arrest, particularly in patients with allergic constitutions, a history of benzodiazepine allergy, or concomitant asthma, where risk stratification is higher (Lee and Kim, 2024). In clinical practice, a detailed allergy history must be obtained prior to administration. For high-risk patients, prophylactic infusion of 50 mL saline is recommended before remimazolam administration. During the initial infusion phase (first 5 min), the infusion rate should be slowed, with close monitoring of blood pressure and skin reactions. Should allergic symptoms occur, infusion must be immediately discontinued, and emergency measures such as epinephrine administration, fluid resuscitation, and external chest compressions should be initiated. Additionally, Yamamoto et al. reported a case where a patient fell asleep again after flumazenil reversed the sedative effects of remimazolam (Nakai et al., 2024). A possible reason is that flumazenil’s mechanism of action against benzodiazepines is based solely on competitive antagonism. Specifically, as plasma flumazenil concentrations decrease, the unmetabolized active components of remimazolam can rebind to benzodiazepine receptors, leading to a recurrence of sedative effects. Based on this, the following recommendations are provided for clinical practice: When using flumazenil to reverse remazolam sedation, the initial dose should be 0.2 mg administered intravenously. If sedation does not improve after 2 min of observation, an additional 0.1 mg may be administered (maximum total dose not exceeding 1 mg). Following reversal, continuously monitor the patient’s level of consciousness, respiration, and blood pressure for at least 2 h. Particularly for elderly patients or those with hepatic or renal impairment, where remimazolam metabolism is slowed, extend monitoring to 4 h. Avoid allowing patients to leave the monitored area unattended. (Masui, 2023). Mao et al. reported an incident involving three patients who failed to achieve adequate sedation despite receiving high doses of remimazolam (Miyanishi et al., 2022). Therefore, in clinical practice, when cumulative remimazolam doses reach 15 mg without achieving effective sedation, prompt assessment of individual patient differences is essential. Avoid blindly increasing doses to prevent cumulative adverse reactions. It is recommended to switch to alternative sedatives or adopt combination therapy regimens. In summary, based on these adverse event reports, clinicians must exercise vigilant monitoring of vital signs when administering remimazolam for sedation. When faced with potential adverse reactions or events, physicians should promptly administer appropriate pharmacological interventions and implement life-support measures as necessary to ensure patient safety.

## 5 Discussion

Based on current research findings, as a novel benzodiazepine sedative, remimazolam has gained limited application in routine procedural sedation due to its rapid onset, swift metabolism, and lower risk of respiratory and circulatory depression. Its safety and efficacy in specific scenarios have been supported by some studies. However, unresolved issues and potential risks must also be acknowledged. First, the lack of long-term safety data represents a critical gap that cannot be overlooked. Existing clinical evidence primarily stems from studies involving short-to-medium-term use. There remains an insufficient body of evidence-based medical data regarding the safety of continuous administration beyond several weeks, potential dependence associated with long-term use, withdrawal symptoms, and effects on neurocognitive function. This uncertainty complicates its application in chronic disease sedation or scenarios requiring repeated sedation. Second, debates over its cost-effectiveness remain inadequately explored. For instance, compared to established, low-cost sedatives like propofol, whether remimazolam’s higher pricing can offset costs through reduced adverse reactions and shorter recovery times—thereby lowering overall healthcare expenditures—lacks robust support from large-scale, multicenter health economics studies. Additionally, regional disparities in accessibility may limit equitable realization of its clinical value. Currently, remimazolam remains unapproved in some countries and regions. Even in approved areas, variations in drug supply across different healthcare levels and health insurance reimbursement policies may limit its availability to only a small number of patients, hindering its adoption as a universally accessible sedation option. Finally, concerns regarding its use in specific populations and the associated learning curve require attention. For special populations such as elderly patients, those with hepatic or renal impairment, and obese patients, current dosage recommendations are largely derived from small-sample studies, resulting in significant individual variability. Clinicians must gradually optimize treatment regimens through accumulated experience, presenting a learning curve. Furthermore, safety data for populations like pregnant women and children remain severely inadequate, limiting the expansion of its applicable scope.

Overall, as a novel sedative, remimazolam demonstrates certain application potential in clinical sedation practices. However, current research evidence remains significantly limited. Overemphasizing its advantages while overlooking potential risks and unresolved issues may misguide clinical decision-making. Future efforts should include more high-quality long-term follow-up studies, health economics research, and population-specific drug studies to clarify its appropriate indications and risk-benefit ratio. Concurrently, optimizing drug supply policies and improving medical insurance reimbursement mechanisms are essential to narrow accessibility gaps. Only by adopting a more critical and neutral perspective on its clinical value can we promote the rational use of remimazolam, ultimately benefiting patients.
